# Hysteria in Childhood

**Published:** 1879-11

**Authors:** Herman Mynter


### TRANSLATIONS.

### HYSTERIA IN CHILDHOOD.

TRANSLATED FROM DANISH BY HERMAN MYNTER, M. D.

PROFESSOR GAEDEKEN, of the University of Copenhagen, has lately published a paper upon this subject which we consider of interest for our readers.

The writer gives first some general remarks upon our want of knowledge of the pathology of hysteria, and consequently the difficulty in classifying these diseases, especially as the name is used to signify certain forms of insanity. Formerly it was supposed that this disease had a certain causal connection with the female sexual organs, and would only be found in women; and furthermore only when the sexual organs are developed. Late experience has shown that hysteria, in fully developed form, may be found in nervous men, and occasionally in children of both sexes. The symptoms in children, although often very violent and apparently of very serious nature, yield in a remarkable manner to moral treatment. The cases mentioned are seventeen in all, of which four were girls, thirteen boys. Our space allows us to publish only the most interesting.

1. M. H., a girl 10 years of age, entered the City Hospital in December, 1878; no hereditary predisposition; the patient gives an impression of being a spoiled child; her manners are conscious, affected and silly; eight days previous, she had, without any known cause, attacks of convulsions, commencing as a convulsive tremor of the whole body, little by little increasing to violent clonic spasms; the attacks last about half a minute, and occur as often as a hundred times in 24 hours, day and night; she is but partly conscious during the attack, after which she is immediately well, and continues the sentence at the point at which it was broken off. The family is grieved and anxious on account of her condition. It is also noted that she is well nourished, very affected and also occupied with her disease ; likes to speak and describe it ; if we desire to witness an attack, she has one immediately, lasting 25 to 30 seconds, consisting of strong clonic spasms in muscles of the face, body and extremities ; it is evident that she takes good care not to hurt herself during the spasms ;—left alone, she slept well the first night ; in the course of the following day she had about a hundred attacks, which were perfectly ignored ; at the next visit she was severely spoken to during an attack which was perfectly interrupted by the fright. She was then told that she should stop the spasms ; that she was able to control them if she would, and that visits would not be permitted to her until the spasms stopped ; during the day but a few and feeble spasms, after that none ; seven days afterwards she was discharged cured.

2. M. K., a boy eight years of age ; no hereditary predisposition, but a sister is hysterical ; had a fall when ten months old, from the third story, fracturing the thigh and receiving some contusions of the head ; for a year has suffered frequently from headache and vomiting, sometimes accompanied with delirium ; suffered for ten weeks from protracted and strong attacks of convulsions, occurring especially during evening and night, as often as ten times in twenty-four hours ; they commence with crying, the patient becomes stiff in the neck, then in the whole body, with convulsive spasms in the extremities, froths at the mouth, etc. He is very irritable and exacting, compels the mother to carry him around during the attacks, beats and kicks her in his spasms, and tries to bite her nose ; has attended school till lately, where he was clever and behaved himself well. The mother is a widow, very good natured, weak and irresolute, and unable to make herself respected by the children. The patient entered the hospital in November, 1875, was healthy-looking and without any traces of disease. He answers questions rationally, speaks with a certain pride of his spasms, which he paints with bright colors : " First, I get as stiff in the neck and back as a stick, then black as coal in the face, froth at the mouth, and want to bite everybody near me."

He was greatly astonished and indignant, the first evening, in receiving the injunction not to have another attack, as he would be put in a cell, a place painted to him in very dark colors. He was thereafter well for three weeks, and had but one attack at the first visit of the mother, who was immediately sent away, and after a serious menace the attack disappeared. Nothing heard from him since he left the hospital.

3. C. A., a girl nine years of age, inmate of a public institution, entered the hospital in March, 1878; no information of hereditary predisposition; has been four years in the institution; was always perfectly well, but is described as a quiet and dispirited child, shy and spoiled; will not play with the other children. Seven months previous she had an attack of chorea, lasting one month and resulting in a paresis of both lower extremities, so that she was unable to walk, and during half a year crept around on hands and knees. The treatment consisted in iron, baths, etc., but without any result. For a month she has vomited after eating anything she dislikes, and it has been necessary to cook separate dishes for her; she does not vomit if served with what she likes; has lost flesh, complains of feeling tired and weak; the urine passes involuntarily every moment.

At the hospital it is noted that she is a pale, thin and tiny child, with whimpering, dispirited manners; she cried and desired to go home again. At the examination the first evening she was able to move all muscles of the lower extremities perfectly well while laying in bed, and with full and almost normal power; nevertheless she asserts that she cannot stand upon her feet; she was immediately taken out of bed, put on the floor and peremptorily ordered to stand; after a few awkward attempts to tumble and fall, she finally stood, as she found that no one would support her, commenced thereafter to walk, and walked at last without support through the whole corridor; was ordered iron and strong nourishment; she surprised very much, the next day, visitors from her home, by running around quickly in the hall, slept and ate well, became soon confident and had natural childish manners. During three weeks she had no symptom of disease whatever, and was then discharged.

4. J. V., 13 years of age, pupil in a college outside Copenhagen; no hereditary predisposition; has had the common diseases of childhood; always been well. In August, 1876, he left his parents' home and entered the college, where he showed himself a clever and smart boy. After four days he was taken sick, and was confined to his bed for three weeks with what was considered to be a gastric trouble; the convalescence was exceedingly slow; complained now and then of headache; was for a long time excused from study, and at last sent home to remain till after vacation. At home he soon recovered and returned to school again in January, 1877. After scarcely four weeks the disease commenced again, with pains in both knees, considered to be rheumatism and resisting all treatment. He complained of heaviness and pain in the head, sleeplessness, nausea, weakness and constipation; the pupils were a little dilated and unequal, the pulse irregular (60 to 65); complained furthermore of hallucinations; became touchy and difficult to manage; domineered over his attendants; cannot eat anything except what he likes, and demands the most absurd things; during the night he writes the announcement of his own death and funeral with mourning verses, frightens his nurse by making her believe that he has a pistol in his bed, with which he will shoot her, complains now of this, now of that, and of hallucinations of the most remarkable nature; keeps his bed all the time, and complains of pains everywhere. A physician who was consulted considered him insane, and all his attendants believed him seriously ill. This comprised the record of the case.

Taught by former experience, I advised him to be sent to the hospital, and promised the symptoms would soon disappear. They acceded to my advice with great hesitation, and were so apprehensive over the trial, that, during the journey, they sent a dispatch to that effect; the patient, however, endured the journey well. On arriving at the hospital I spoke seriously with him.

He was pale and thin, and had a suppurating issue in the neck. The examination did not show any disease; the pulse was 72, a little irregular; ordered iron and arsenic in increasing quantity, and the issue to be allowed to heal. He ate and slept well, presented no symptoms of disease whatever till he was discharged, after which time he remained some time in Copenhagen and was perfectly well. Against my advice he was sent back to school, where the former symptoms, in a little while, commenced again. Consulted again, I advised to ignore his disease, and have since heard nothing about him.

The cases mentioned above, Professor Gaedeken says, are similar to those which are described by different authors as hysteria in childhood. We may, to be sure, assert that there was nothing whatever the matter with some of these children, that they have been naughty and spoiled at home, but as soon as they entered the hospital became well-behaved and did not show any symptom of disease (see case No. 2 and 4). It is not an unfrequent occurrence that boys, of 15 or 16 years of age, will not work, and therefore make their parents, especially their mothers, believe that they must stay at home, that they are sick, etc., but such cases are easily recognized, and are not called hysteria. But it can scarcely be believed that a little boy of eight years (see case No. 2) should for ten weeks disturb his own and his mother's sleep, simply to be carried on her arm and have the pleasure to kick her and bite her nose. Such an act would suppose an amount of energy which such a child could not display. On the other hand, Professor Gaedeken acknowledges that hysterical symptoms especially are found in spoiled nervous children, that they may come by imitating a hysterical sister, or be reared by a foolish compliance of the parents, and that the symptoms may get worse and worse, because the child willingly gives up to its sufferings and considers that its convulsions are interesting and of remarkable occurrence. Under such circumstances, especially if the nutrition is feeble, dangerous symptoms may be developed while we may see, especially in the commencement, that the children if taken away from home and their disease ignored, are able to control their symptoms, which surely may be considered unnatural in childhood, and for that reason are more easily forgotten and subdued than in older children.

